# Photocatalytic Degradation of DIPA Using Bimetallic Cu-Ni/TiO_**2**_ Photocatalyst under Visible Light Irradiation

**DOI:** 10.1155/2014/342020

**Published:** 2014-06-29

**Authors:** Nadia Riaz, Mohamad Azmi Bustam, Fai Kait Chong, Zakaria B. Man, Muhammad Saqib Khan, Azmi M. Shariff

**Affiliations:** ^1^Chemical Engineering Department, Universiti Teknologi PETRONAS, 31750 Tronoh, Malaysia; ^2^COMSATS Institute of Information Technology, Tobe Camp, University Road, Abbottabad 22060, Pakistan; ^3^Fundamental & Applied Sciences Department, Universiti Teknologi PETRONAS, 31750 Tronoh, Malaysia

## Abstract

Bimetallic Cu-Ni/TiO_2_ photocatalysts were synthesized using wet impregnation (WI) method with TiO_2_ (Degussa-P25) as support and calcined at different temperatures (180, 200, and 300°C) for the photodegradation of DIPA under visible light. The photocatalysts were characterized using TGA, FESEM, UV-Vis diffuse reflectance spectroscopy, fourier transform infrared spectroscopy (FTIR) and temperature programmed reduction (TPR). The results from the photodegradation experiments revealed that the Cu-Ni/TiO_2_ photocatalysts exhibited much higher photocatalytic activities compared to bare TiO_2_. It was found that photocatalyst calcined at 200°C had the highest photocatalyst activities with highest chemical oxygen demand (COD) removal (86.82%). According to the structural and surface analysis, the enhanced photocatalytic activity could be attributed to its strong absorption into the visible region and high metal dispersion.

## 1. Introduction

Semiconductor photocatalysis has been investigated extensively for light-stimulated degradation of pollutants, particularly for complete destruction of toxic and nonbiodegradable compounds into carbon dioxide and inorganic constituents [1]. The fundamentals of semiconductor photocatalysis and its application to the removal of chemical pollutants have been extensively reviewed [2]. Several semiconductors exhibit bandgap energies suitable for photocatalytic degradation of contaminants. Among the photocatalysts applied, titanium dioxide is one of the most widely employed photocatalytic semiconducting materials because of the peculiarities of chemical inertness, nonphotocorrosion, low cost, and nontoxicity. Doping semiconductors with various metal ions, composite semiconductors, deposition of transition metals, and oxygen reduction catalysts can be employed to enhance photocatalytic efficiency [3]. The photocatalytic process is characterized by the production of ^∙^OH radicals which are able to oxidize and mineralize organic compounds [4].

However, a serious limitation for the application of TiO_2_ semiconductor in heterogeneous photocatalysis is the requirement of UV light to promote the electron transference process due to its wide bandgap (about 3.2 eV). For the fabrication of visible light active photocatalysts, the modification of semiconductors by the addition of transition metals and nonmetal doping are the commonly adopted ways [5–7]. Modification of titania by doping using transition metals has often been used to enhance its photocatalytic activity and to extend its absorption wavelength from the UV to visible region [8]. The use of Cu and Ni as bimetallic catalysts supported on different semiconductor materials has been reported as the effective method to improve the efficiency of various reactions like carbon dioxide hydrogenation [9], steam reforming of methane [10], liquid-phase glycerol hydrogenolysis by formic acid over Ni-Cu/Al_2_O_3_ catalysts [11], decomposition of methane over Ni-SiO_2_ and Ni-Cu-SiO_2_ catalysts [12], photocatalytic reduction of nitrate [13], azo dye degradation using Cu-Zn/TiO_2_ [8], Ni/TiO_2_ [14] and Cu-Fe/TiO_2_ [15] for methyl orange degradation, and Cu/TiO_2_ for Orange II degradation [16].

The objective of the present work is to investigate the photocatalytic degradation of diisopropanolamine (DIPA) in the presence of Cu-Ni/TiO_2_ photocatalyst under visible light source. The introduction of Cu and Ni was with the intention to reduce the bandgap of the photocatalyst for enhanced visible light absorption. In order to obtain a better understanding, we investigated the influence of various parameters that may affect the photodegradation of DIPA.

## 2. Experimental

### 2.1. Materials

Copper nitrate trihydrate, Cu(NO_3_)_2_ *·*3H_2_O, and nickel nitrate hexahydrate, Ni(NO_3_)_2_ *·*6H_2_O (Acros brand > 98% purity), were used as dopant metal salts. Titanium dioxide, TiO_2_ (Degussa-P25 80% anatase, 20% rutile), was used as the support which also acts as the semiconductor in photocatalysis. Diisopropanolamine (DIPA) (Merck, Germany) was used as the model alkanolamine for photocatalytic degradation study. All chemicals were used as received.

### 2.2. Photocatalyst Preparation

Bimetallic Cu-Ni/TiO_2_ photocatalysts with 10 wt% total metal loading and 9Cu : 1Ni mass composition were prepared using wet impregnation (WI) method with TiO_2_ as support. 9Cu : 1Ni mass composition and 10 wt% metal loading were selected based on the results reported in [18]. To prepare photocatalysts using WI method, support was added into the metal salt solution. The suspension was stirred for 1 hour before the solvent was evaporated in a water bath at 80°C until a thick paste was obtained. This paste was then dried in an oven at 120°C for 18 hours. The dried photocatalyst was ground with a mortar and pestle, kept in air-tight glass bottle (to avoid moisture) as raw photocatalyst, and stored in a desiccator at room temperature prior to calcination.

### 2.3. Characterization

The photocatalysts were characterized using different characterization techniques to understand the chemical and physical properties and then to relate these properties to their photocatalytic performance. In order to estimate suitable calcinations temperatures for the raw photocatalysts, thermal gravimetric analysis (TGA) was carried out using Perkin Elmer (Pyris 1 TGA) instrument. The morphology of the photocatalysts such as crystallite particle shape and size distribution was analyzed using FESEM (Supra55VP). Reflectance spectrums were recorded at 190–800 nm wavelength using DR-UV-Vis spectrophotometer (Shimadzu Lambda 900). Barium sulphate (BaSO_4_) powder was used as a standard, an internal reference. Fourier transform infrared spectroscopy (FTIR, Shimadzu FTIR-8400S) analyses were carried out to identify species present in the photocatalysts. A small amount of photocatalyst was grained together with 50 mg of IR-grade KBr and pressed into pellet using a hydraulic hand press. Later the pellet was placed in the sample holder and scanned at 4000 cm^−1^ to 400 cm^−1^. KBr was used as the background file. The temperature programmed reduction (TPR) analyses were conducted in order to determine reducibility of the photocatalysts and metal dispersion in photocatalysts using Thermo Finnigan equipment (TPDRO 1100). Prior to reduction, the sample was pretreated under nitrogen at 110°C with a flow rate of 20 mL min^−1^ and ramp rate of 10°C min^−1^ and finally holding at 110°C for 30 minutes to eliminate moisture before cooling to room temperature. TPR analysis was carried out in 5% H_2_ in N_2_ with a flow rate of 20 mL min^−1^. Samples were heated with a ramp rate of 20°C min^−1^ from 40°C to 500°C and holding at 500°C for 10 minutes. The reduction profile was shown in a plot of hydrogen consumption as a function of linearity temperature.

### 2.4. Measurements of Photocatalytic Activities

Photocatalytic activity of the prepared photocatalysts was evaluated by monitoring the photocatalytic degradation of alkanolamine in aqueous solution under visible light irradiation. For a typical experiment, photocatalysts were weighed and mixed with distilled water and then ultrasonicated for 5 min using sonicator followed by the addition of DIPA solution (final concentration of 100 ppm, photocatalyst loading of 1 g*·*L^−1^, and total volume of 100 mL). The suspension was stirred in the dark for 30 minutes and later this suspension was illuminated for 1 h using the 500 W halogen lamp as the visible light source. Reaction study was carried out at atmospheric pressure and room temperature (at 25 ± 1°C) that was controlled by continuous cooling air. The apparatus used for reaction study has been described elsewhere [14]. Samples were collected at different intervals of time (min) and were immediately centrifuged twice to remove suspended solid photocatalyst and monitored for further analysis. For the photodegradation experiments at different pH values, the initial pH of the reaction suspension was adjusted by the addition of NaOH or HCl solutions.

The degradation of alkanolamine was measured by chemical oxygen demand (COD) using Hach UV-Vis spectrophotometer (DR 39000) to measure the COD/TOC concentration (mg*·*L^−1^) for the reaction samples. In order to determine the mineralization efficiency of photocatalyst, COD removal (%) was calculated as follows:

(1) $$\begin{array}{c} \text{COD}\;\text{removal}\;\left(\%\right)=\left(\frac{{\text{COD}}_{0}-{\text{COD}}_{t}}{{\text{COD}}_{0}}\right)\times 100, \end{array}$$

where COD_0_ is the initial COD in ppm and COD_*t*_ is the final COD concentration in ppm at different time intervals during reaction.

## 3. Results and Discussion

### 3.1. Photocatalyst Preparation and Characterization

Bimetallic Cu-Ni/TiO_2_ photocatalysts were prepared using wet impregnation (WI) method with TiO_2_ as support. Total metal loading (10 wt%) and Cu : Ni mass composition (9Cu-1Ni) were fixed based on the optimization results reported by our research group [18]. Prepared photocatalysts were characterized using different characterization techniques in order to relate the physicochemical properties with the photocatalyst performance.

Results from TGA were reported as thermograms as shown in our previous work [17], which are plots of the relative weight of the photocatalyst versus temperature. Based on the TGA results, calcination was conducted at selected temperatures for 1 h duration. The calcined photocatalysts were given denotation: *x*Cu-*y*Ni-*T* where “*x*” and “*y*” represent the mass composition of Cu and Ni, respectively, with *“x”* + *“y”* = 10, while “*T*” represents calcination temperature in °C. Photocatalysts were calcined at three different calcination temperatures: 180, 200, and 300°C.

**Figure 1:**
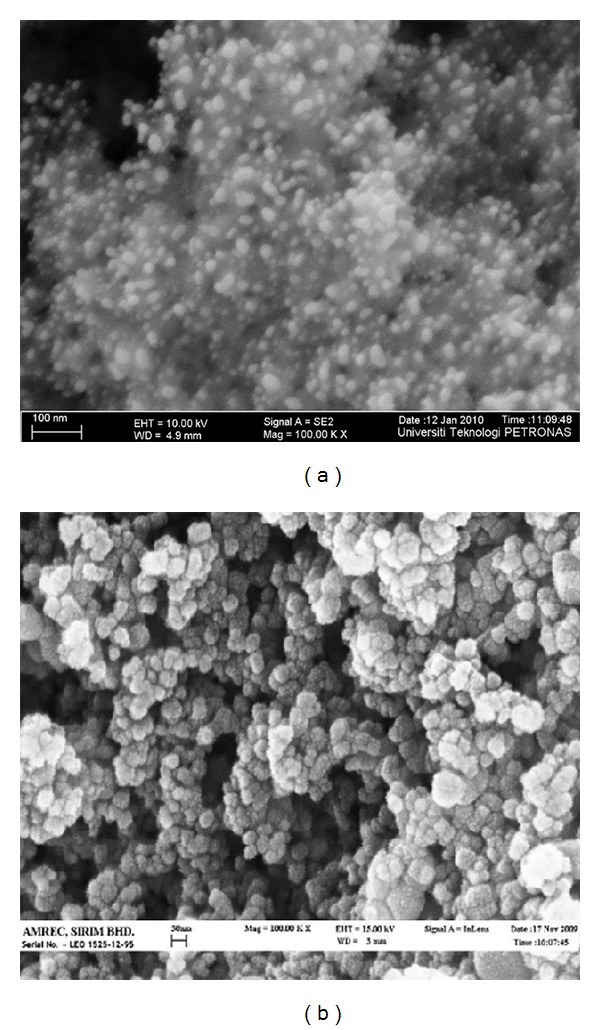
FESEM micrograph of (a) bare TiO_2_ and (b) Cu-Ni/TiO_2_ photocatalysts (at magnification 100KX) [17].

FESEM (field-emission scanning electron microscopy) micrographs for the bare TiO_2_ and photocatalysts calcined at 200°C are presented in Figure 1. The micrographs of the Cu-Ni/TiO_2_ photocatalysts clearly depict uniform distribution with spherical morphologies and slight agglomeration ranging from 11 to 40 nm. Comparing micrographs of bare TiO_2_ with bimetallic it is clear that addition of Cu-Ni to bare TiO_2_ can help to enhance the particle shape and size. The photocatalysts samples were partly composed of clusters containing composite nanoparticles adhering to each other. The agglomeration might be due to sintering during calcination process. From the EDX spectrum it was observed that metal oxides were well dispersed over the TiO_2_ support.

**Figure 2:**
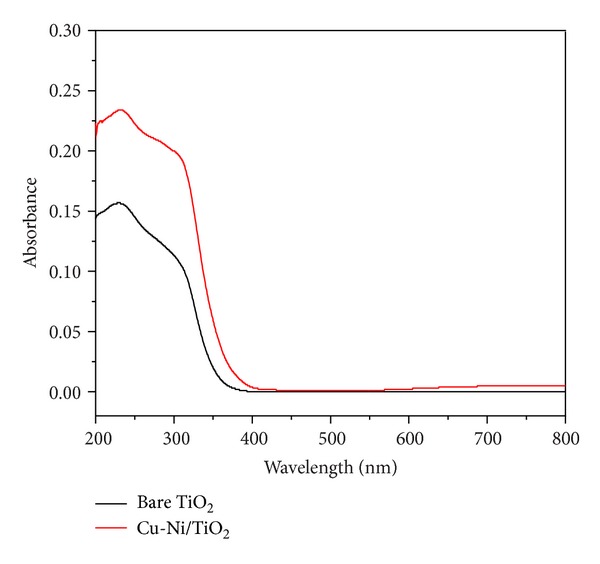
Absorption spectra for bare TiO_2_ and bimetallic Cu-Ni/TiO_2_ photocatalysts.

Modification with metal has shifted the absorption spectrum of TiO_2_ into visible region. The diffuse reflectance UV-visible (DR-UV-Vis) spectra of bare TiO_2_ (Figure 2) showed absorption peaks ranging from 190 nm to 400 nm, similar to that observed in our previous studies [18–20]. The shift in the absorption spectrum has been observed for the synthesized photocatalysts. Impregnation method has also reported previously that this method can also shift the absorption spectrum of TiO_2_ [21]. The shift might not possibly be caused by the change in the bandgap but rather might be by the impurity of energy level as the metal is spread on the surface of TiO_2_ photocatalyst.

Results of physical characterization by diffuse reflectance UV-visible (DR-UV-Vis) spectra and FESEM indicate that the copper and nickel metal were highly dispersed and interacted with the TiO_2_ support. High dispersion and low bandgap energy together play synergetic effect for photodegradation performance of the photocatalysts under visible light irradiation. The lower bandgap energy of bimetallic photocatalysts compared to that of bare TiO_2_ and monometallics also becomes a reason of its higher activity.

**Figure 3:**
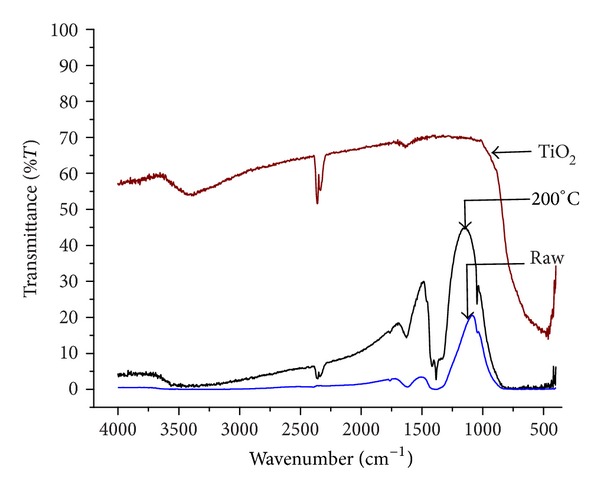
FTIR spectra of bare TiO_2_ and Cu-Ni/TiO_2_ photocatalysts (raw and calcined at 200°C).

FTIR spectra of bare TiO_2_ and raw and calcined photocatalysts (10 wt% Cu-1Ni/TiO_2_-200) are shown in Figure 3. Several absorption peaks were observed. The broad band around 3400 cm^−1^ was attributed to O–H stretching and the peak near 1600 cm^−1^ was attributed to H–O–H bending and related to physically absorbed moisture [19, 22]. The IR band observed from 400 to 900 cm^−1^ corresponds to the Ti–O stretching vibrations [23–25]. The intense peak at 1384 cm^−1^ was ascribed to nitrate (NO_3_^−^) group which is present in all the spectra. The presence of nitrate band was also observed by Mohan [26] and Li and Inui [27]. They mentioned that the nitrate will always be present when nitrate salts are used as precursors. Possible assignment of the peaks observed in FTIR spectra of bare TiO_2_ and Cu : Ni/TiO_2_ photocatalysts is shown in Table 1.

**Figure 4:**
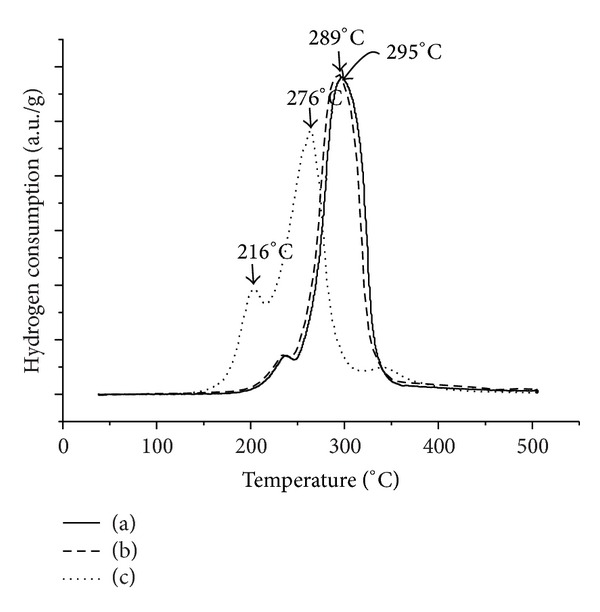
The TPR profiles of Cu-Ni/TiO_2_ photocatalysts calcined at different temperatures: (a) 200, (b) 180, and (c) 300°C.

Temperature programmed reduction (TPR) was used in order to characterize the Cu-Ni/TiO_2_ photocatalyst with respect to the type of metal oxide species present, either copper oxide, nickel oxide, or copper-nickel mixed oxide, and the degree of interaction of the oxides with TiO_2_ support [19]. The summary of the amount of hydrogen consumed and reduction temperature of different photocatalysts is shown in Table 2. The TPR profiles of the bimetallic Cu-Ni/TiO_2_ photocatalysts with different calcination temperatures are presented in Figure 4. The higher reduction peaks at 295°C were ascribed to bulk CuO phases that include large clusters and bulk CuO. The reduction profile of bimetallic Cu-Ni/TiO_2_-200 showed a shoulder around 220–240°C and one main reduction peak at 289°C, which might be attributed to the reduction of Cu-Ni mixed oxide instead of individual oxide. Higher reduction temperatures are attributed to the reduction of NiO with strong interaction with TiO_2_ [28]. The distinct peak observed at 289°C for 9Cu : 1Ni-180 might be attributed to the reduction of Cu-Ni mixed oxide instead of individual oxide [29]. It was also found that the presence of Cu lowered the reduction temperature of Ni. Li et al. [30] observed the same behavior as the bimetallic Cu-Ni/TiO_2_. The addition of Cu enhanced the reduction of Ni; thereby, it can be concluded that the reducibility of bimetallic Cu-Ni is controlled by the amount of Cu. However, the presence of Cu-, Ni-, and mixed Cu-Ni species was not detected due to high dispersion of the metal particle on TiO_2_. The TPR profile of photocatalyst is in good agreement with the results shown by FESEM that the Cu- species was highly dispersed on TiO_2_ photocatalyst.

### 3.2. Photocatalytic Activity of Cu-Ni/TiO_2_ Photocatalysts

#### 3.2.1. Effect of Irradiation Time

**Figure 5:**
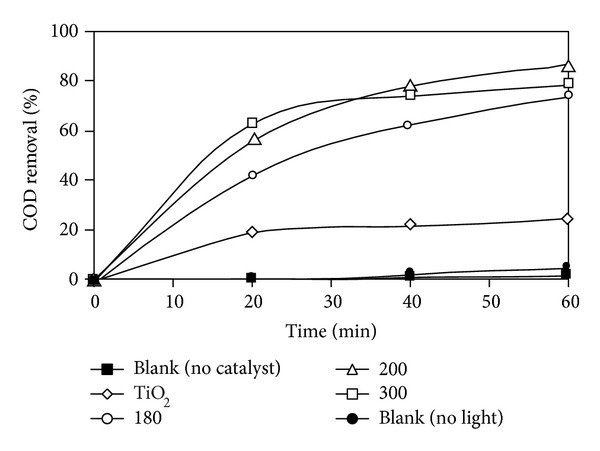
Photocatalytic degradation of DIPA under visible light using bimetallic Cu-Ni/TiO_2_ photocatalysts (calcination temperatures = 180, 200, and 300°C; DIPA concentration = 100 ppm; reaction temperature = room temperature 23 ± 1; reaction volume = 100 mL; irradiation duration = 60 min; light source = 500 W halogen lamp; photocatalyst loading = 1 g*·*L^−1^).

Photocatalytic degradation of DIPA under visible light using bimetallic Cu-Ni/TiO_2_ photocatalysts is displayed in Figure 5. COD removal using Cu-Ni/TiO_2_ photocatalysts calcined at different calcination temperatures was calculated in terms of % COD removal. It is evident that the percentage of mineralization increases with irradiation time for photocatalysts calcined at different temperatures. The highest COD removal (%) was for photocatalyst calcined at 200°C. However, for photocatalyst calcined at 180 and 300°C and bare TiO_2_, degradation was comparatively very slow with 74.16, 78.72, and 24.47% COD removal.

#### 3.2.2. Effect of pH

**Figure 6:**
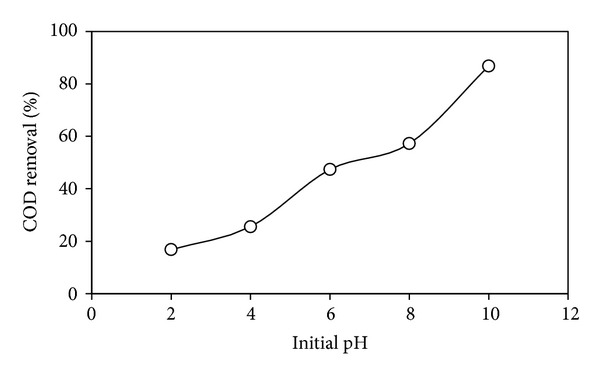
Effect of pH on photodegradation of DIPA using Cu-Ni/TiO_2_ photocatalysts (photocatalysts loading = 1 g*·*L^−1^; DIPA concentration = 100 ppm; irradiation time = 1 h).

The pH influences the characteristics of the photocatalyst surface charge, so pH of the solution is a significant parameter in performing the reaction on surface of TiO_2_ particles for the photocatalytic degradation of organic pollutants [31]. The photodegradation of DIPA in the presence of 10 wt% Cu-Ni/TiO_2_ photocatalysts is in the range between 2 and 12 as shown in Figure 6. From the previous studies, it is very clear that pH of a solution influences the surface charges of TiO_2_ affecting the interfacial electron transfer and the photoredox process [32]. The point of zero charge (PZC) of TiO_2_ is 6.25 and its surface is predominately positively charged below PZC and negatively charged above PZC. Alkanolamines are mostly protonated at pH 9 or below [31, 33]. From Figure 6, it is clear that the photodegradation increases with the increase in pH. This might be attributed to the increase in the number of OH^−^ ions at the surface of TiO_2_; hence, at acidic pH values, the particle surface is positively charged and active species like hydroxyl radical may not be adsorbed onto the positively charged TiO_2_ surface, resulting in a decreased photodegradation. Results are in agreement with the previous studies [31, 33] which reported enhanced photodegradation at alkaline pH.

#### 3.2.3. Effect of Calcination Temperature

**Figure 7:**
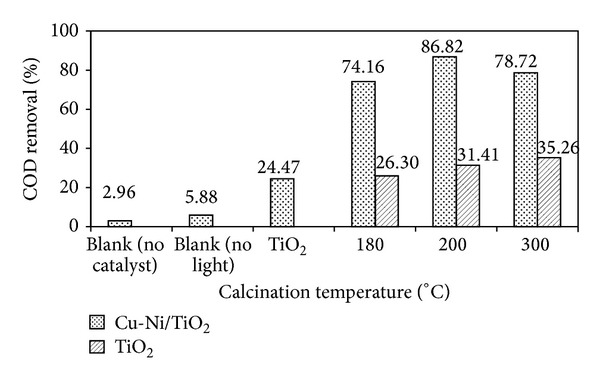
Effect of calcination temperature on photodegradation of DIPA using bare TiO_2_ and Cu-Ni/TiO_2_ photocatalysts (photocatalysts loading = 1 g*·*L^−1^; DIPA concentration = 100 ppm; calcination temperature = 180, 200, and 300°C; irradiation time = 1 h).

Photocatalysts calcined at 180, 200, and 300°C were screened for photodegradation of 100 ppm DIPA under visible light irradiation. The % COD removal for different calcination temperatures was 74.16%, 86.82%, and 78.72% for photocatalysts calcined at 180, 200, and 300°C, respectively, as shown in Figure 7. The highest COD (%) removal was obtained for the photocatalysts calcined at 200°C. The results also indicate that the photocatalytic activity of synthesized titania decreased with the increased calcination temperature. Yu and coresearchers [34] also reported that a significant decrease in the photocatalytic activity of titania nanopowder calcined at higher temperature may be attributed to the growth of particle and result in the reduction of contact area of particles for photocatalytic reaction. The improvement of photocatalytic activity compared with commercial materials can be associated with the combined increase of crystallinity with the preservation of a relatively large surface area based on the existence of mesopores [35].

#### 3.2.4. Effect of Photocatalyst Loading

**Figure 8:**
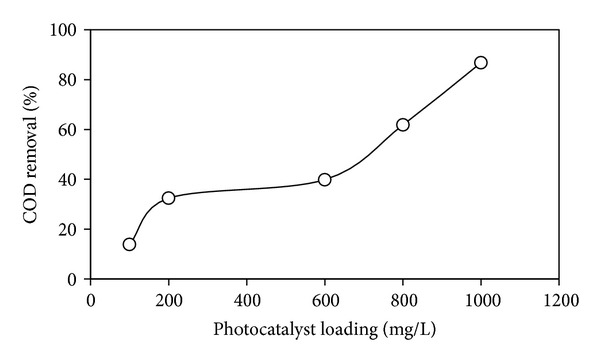
Effect of photocatalyst loading on photodegradation of DIPA using Cu-Ni/TiO_2_ photocatalysts (photocatalysts loading = 100–1000 g*·*L^−1^; calcination temperature = 200°C; DIPA concentration = 100 ppm; irradiation time = 1 h).

Effect of photocatalyst amount on the photodegradation of DIPA is shown in Figure 8. Photocatalysts loading was in the range of 100–1000 g*·*L^−1^. COD removal (%) was increased with increasing photocatalyst amount, indicating the availability of active sites on the photocatalyst surface for the adsorption of pollutant.

#### 3.2.5. Effect of Initial DIPA Concentration

**Figure 9:**
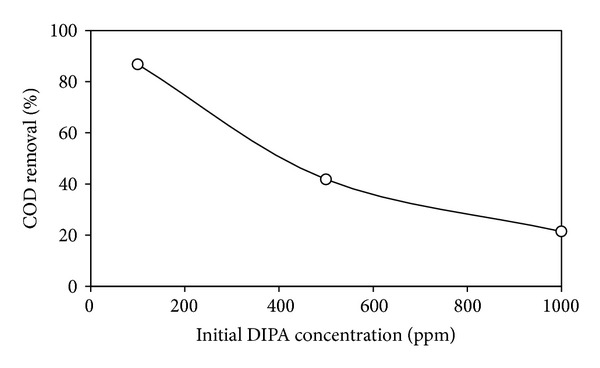
Effect of initial DIPA concentration on % COD removal using Cu-Ni/TiO_2_ photocatalysts (photocatalysts loading = 1 g*·*L^−1^; calcination temperature = 200°C; DIPA concentration = 100–1000 ppm; irradiation time = 1 h).

Effect of initial DIPA concentration that was studied by fixing the photocatalyst amount (based on the optimum photocatalyst loading 1 g*·*L^−1^) on the photodegradation of DIPA is shown in Figure 9. COD removal (%) decreased with increasing initial DIPA concentration (100–100 ppm) indicating that as the pollutant concentration increases more organic substances are adsorbed on the surface of TiO_2_, whereas less number of photons are available to reach the catalyst surface and therefore less ^*∙*^OH radicals are formed, thus causing an inhibition in degradation percentage [36].

#### 3.2.6. Effect of Cu-Ni Codeposition

**Figure 10:**
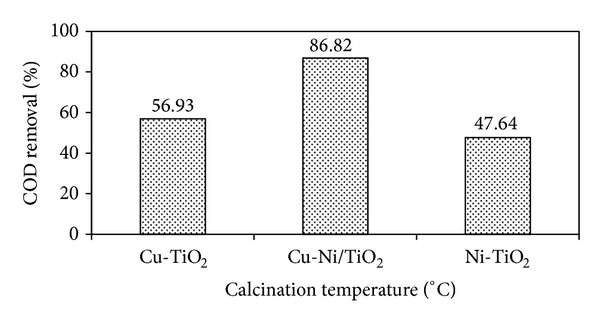
Effect of Cu-Ni codeposition on % COD removal using Cu/TiO_2_, Ni/TiO_2,_ and Cu-Ni/TiO_2_ photocatalysts (photocatalysts loading = 1 g*·*L^−1^; calcination temperature = 200°C; DIPA concentration = 100 ppm; irradiation time = 1 h).

The photocatalytic activities of the 10 wt% monometallic and bimetallic Cu, Ni, and Cu-Ni photocatalysts are presented in Figure 10. From the results, it is clear that the ratio of metal content in bimetallic catalysts plays a significant role in photocatalytic activity. The optimum COD removal (%) was obtained from bimetallic Cu-Ni/TiO_2_ photocatalysts with 86.82%. For the monometallic photocatalysts, Cu/TiO_2_ displayed better performance compared to Ni/TiO_2_. However, their performance was lower than that of bimetallic Cu-Ni/TiO_2_ and higher compared to that of bare TiO_2_. In present study it was found that the addition of small amount of Ni enhanced the activity of bimetallic photocatalyst compared to those of monometallics; this might be due to synergetic effect of two different metals [37]. The presence of Cu and Ni plays a significant role in reduction of electron hole recombination by trapping electron and hole simultaneously. In particular Ni can enhance the hole trap thus retarded recombination reaction [18].

## 4. Conclusions

Surface modification of TiO_2_ with copper and nickel had been successfully carried out using wet impregnation (WI) method with TiO_2_ as support. Compared with the commercial reference, bare TiO_2_ P25, the diffuse reflectance spectra suggest that the photoabsorption of Cu-Ni/TiO_2_ photocatalysts is extended to the visible region. It is assumed that these Cu-Ni photocatalysts have induced impurity levels between the conduction and valence band of TiO_2_, leading to narrower bandgap and enhancing the visible light absorption. The effect of process parameters such as irradiation duration (1 h), initial pH (pH range of 2–12), photocatalyst calcination temperature (180, 200, and 300°C), photocatalyst loading (0.1–1 g*·*L^−1^), and initial DIPA concentration (100–100 ppm) has been investigated. The photocatalyst activity is directly related to photocatalysts loading suggesting that, for highly concentrated wastewater, dilution is a necessary step prior to the photodegradation step. Based on the results from the present study, the as-prepared highly active Cu-Ni/TiO_2_ photocatalysts may have a great potential for photocatalytic water purification, particularly the elimination of toxic aromatic compounds under simulated solar light irradiation.

## Figures and Tables

**Table 1:** Assignment of absorption peaks observed in FTIR spectra of bare TiO_2 _and Cu-Ni/TiO_2_ photocatalysts.

Peaks (cm^−1^)	Possible assignment	Related process occurring
1600	H–O–H bending	Physically adsorbed moisture
3400	O–H stretching of hydroxyl group	
1384	NO_3_^−^ anion	Presence of nitrate

**Table 2:** Summary of the hydrogen consumption and the reduction temperature of the photocatalysts.

Photocatalyst	Reduction peak (°C)	Amount of hydrogen consumed (*μ*mol*·*g^−1^)
180°C	295	1877.0
	361	1939.9

200°C	289	1879.3
	357	1938.2

300°C	216	345.79
	276	1436.2
	355	158.98
